# Tramadol Induced Jerks

**DOI:** 10.7759/cureus.17547

**Published:** 2021-08-29

**Authors:** Waiz Wasey, Imad Aziz, Sharefi Saleh, Naila Manahil, Neha Wasey

**Affiliations:** 1 Family and Community Medicine, Southern Illinois University School of Medicine, Springfield, USA; 2 Family Medicine, Mercy Health System, Beloit, USA; 3 Family Medicine, Ruth Temple Clinic, Los Angeles, USA; 4 Family Medicine, Southern Illinois University, Springfield, USA; 5 General Practice, Shadan Institute of Medical Sciences, Hyderabad, IND

**Keywords:** pain control, tramadol induced jerking, myoclonus, tramadol, opioid use disorders

## Abstract

Myoclonus is a sudden, involuntary jerking of a muscle or a group of muscles. Myoclonus may present in form of a pattern or, sporadically and infrequently. It is usually associated with neurological disorders such as epilepsy, multiple sclerosis or infections, and tumors of the central nervous system. Myoclonus is not commonly known to be caused by tramadol. We present a case of a 59-year-old male who developed myoclonus in the muscles of his trunk, 10 days after initiating tramadol for chronic pain. The myoclonus disappeared after withholding the medication. The purpose of this case report is to make clinicians aware of a rare reversible side effect from the use of tramadol.

## Introduction

Sudden involuntary jerking of a muscle or a group of muscles, in form of a pattern or sporadically, is referred to as Myoclonus [1]. Usually associated with neurological disorders, such as epilepsy, multiple sclerosis or infections, and tumors of the central nervous system, it may be caused by medications as well. Gabapentin and pregabalin are known to induce myoclonus commonly [2]. Tramadol is a synthetic opioid, known to cause side effects such as respiratory depression, serotonin syndrome, seizures [3], syncope, prolonged QT (interval in EKG), and dependency. It is not widely popular that this medicine can induce myoclonus [4] and in rare cases Parkinson's like symptoms [5]. 

## Case presentation

A 59-year-old male presented to the clinic with muscular jerking of his trunk. His past medical history included left cervical radiculopathy, lumbar radiculopathy, and left sacroiliac joint dysfunction. He was initially treated with non-steroidal anti-inflammatory drugs (NSAIDs) and gabapentin for this chronic pain. Due to insufficient pain control, tramadol was added to his pain therapy regimen. Within 10 days following initiation of the medication, he developed muscular jerking of his trunk, which brought him to the clinic. He was started on oral tramadol at a dose of 50mg twice daily. The muscular jerks appeared to occur both at rest and with voluntary movements. They were sporadic and occurred multiple times during the clinical encounter. 

On clinical examination, the cranial nerves were found intact. Reflexes of major nerve pathways were intact. Motor and sensory system examination was unremarkable. Sudden involuntary jerking of his trunk was noted during strength testing on his extremities. Similar movements were noted during history taking as well. The myoclonus was subcortical, focal, and spontaneous. Vitals signs were unremarkable. 

The patient was subjected to a thorough evaluation for uremia, hepatic failure, vitamin deficiencies, thyroid abnormalities, and Wilson’s disease, as well as a consultation with a neurologist. The extensive blood work provided (Table 1) the following information.

**Table 1:** Results of Laboratory Blood Work TSH - Thyroid-stimulating hormone; Sed Rate - Sedimentation rate; Hb - Haemoglobin

Lab Test	Result	Reference Value
Copper	75 µg/dL	69-132 µg/dL
Ceruloplasmin	19 mg/dL	16-31 mg/dL
Ammonia	59 µmol/L	16-54 µmol/L
Vitamin B12	201 pg/mL	180-914 pg/mL
Folate	15.9 ng/mL	4-20 ng/mL
TSH	1.483 mIU/mL	0.340-5.600 mIU/mL
Potassium	3.7 mmol/L	3.5-5.1 mmol/L
Creatinine	1.5 mg/dL	0.7-1.3 mg/dL
Calcium	8.9 mg/dL	8.4-10.4 mg/dL
Magnesium	2.2 mg/dL	1.6-2.6 mg/dL
Sed Rate	7 mm/hr	0-20 mm/hr
Hb	15.5 g/dL	14-18 g/dL

A toxicology screen was negative as well (Table 2), tramadol confirmation was positive. There was no alcohol abuse history found.

**Table 2:** Urine Toxicology Results NDL - Non-detectable levels

Drug	Result
Amphetamines	NDL
Barbiturates	NDL
Benzodiazepines	NDL
Cocaine	NDL
Methadone	NDL
Phencyclidine	NDL
Ethanol	NDL
Marijuana	NDL
Propoxyphene	NDL
Methaqualone	NDL

The patient had a detailed Electroneuromyography (EMG) study of the upper and lower extremities which showed irritability, positive waves, fibrillations, increase in polyphase of the motor unit, and decrease in motor unit potential number firing maximal recruitment pattern in both left cervical (C) 7 vertebra and lumbar (L) 5 vertebra distribution. Other muscle examinations were normal. No evidence of lumbar plexopathy was recorded. The EMG study revealed moderately severe radiculopathy in the left C7. Previous Magnetic Resonance Images (MRI) done were unremarkable except indicating radiculopathy as evident in the EMG.

After consultation with neurology and evaluation of his blood work as well as EMG results, it was suggested that the patient stops taking tramadol. The workup ruled out metabolic causes such as uremia, hepatic failure, vitamin deficiencies, thyroid abnormalities, and Wilson's disease. Imagining studies ruled out neuroinfections, tumors, and anoxic brain injury. EMG did not indicate any root/nerve injury explaining myoclonus of the trunk. He was switched from tramadol to hydrocodone-acetaminophen for pain control, and gabapentin was continued. The patient returned in a week for follow-up after stopping tramadol. His myoclonic jerks improved significantly. On a three-week follow-up, the patient denied any jerking episodes. None were noticed during the clinical encounter as well.

## Discussion

Tramadol is a synthetic opioid widely used for acute and chronic pain. The maximum recommended daily dosing is 400mg per day. Although known for commonly causing side effects such as respiratory depression, serotonin syndrome, seizures [3,6], syncope, prolonged QT, and dependency, it is not widely popular that the synthetic opioid can cause myoclonus [4]. In one study it was reported that 12% of all drug-induced myoclonus was caused by opioids, and 25% of these were due to tramadol [7]. Although the statistic seems significant, myoclonus is not usually listed as a significant side effect on popular platforms such as Epocrates (a medical reference application widely used among medical professionals for clinical decision-making) [8].

Tramadol acts on the mu-opioid receptors and γ-aminobutyric acid receptors. It also inhibits the serotonin reuptake. By inhibiting the γ-aminobutyric acid receptor and increasing serotonin levels, tramadol can induce myoclonus [9]. This is a well defined mechanism of the adverse event. Several cases of myoclonus following intravenous and intrathecal administration of opioids have been documented, but fewer have been reported after oral opioid intake. It is speculated that neuroexcitatory metabolites of opioids are responsible for development of myoclonus. 

In our case, the patient suffered from significant radiculopathy and pain but did not develop any neurological complications or muscular deficits. After starting tramadol, the jerking episodes were evident in about 10 days. After a thorough workup, EMG studies, and a neurological consultation, it was believed to be a drug-induced side effect. This consensus was made after ruling out metabolic causes, such as uremia, hepatic failure, thyroid abnormalities, and vitamin deficiencies through blood work; tumors, neuro infections, and anoxic brain injury through imaging; and root/nerve injuries following EMG studies. 

Tramadol was stopped and pain control was achieved with hydrocodone-acetaminophen. As already discussed, tramadol induces myoclonus by increasing serotonin levels, whereas opioid-induced myoclonus is a result of neuroexcitatory metabolites of opioids, and is unlikely from oral intake. The myoclonus decreased in our patient significantly and completely resolved in about three weeks following cessation of the medicine. As no other cause was found to explain the myoclonus other than stopping tramadol, it was concluded that the adverse effect was medication-induced. 

## Conclusions

Tramadol is a widely used synthetic opioid used for acute and chronic pain control. Many clinicians are aware of the common side effects, such as respiratory depression, serotonin syndrome, seizures, syncope, and prolonged QT. Although documented, tramadol is less widely known to cause myoclonus or the development of parkinsonian symptoms. Clinicians must therefore be aware of these side effects that can develop in their patients, even at therapeutic dosing. 
